# Potential hospital cost-savings attributed to improvements in outcomes for colorectal cancer surgery following self-audit

**DOI:** 10.1186/1471-2482-10-4

**Published:** 2010-01-27

**Authors:** Louisa G Gordon, Andreas Obermair

**Affiliations:** 1 Queensland Institute of Medical Research, Genetics and Population Health Division, PO Royal Brisbane Hospital, Herston Q4029, Brisbane, Australia; 2 Queensland University of Technology, School of Public Health, Kelvin Grove Q4029, Brisbane, Australia; 3 Department of Gynaecology and Oncology, Royal Brisbane and Womens Hospital, Herston, Q4006, Brisbane, Australia

## Abstract

**Background:**

One of the potential benefits of surgical audit is improved hospital cost-efficiencies arising from lower resource consumption associated with fewer adverse events. The aim of this study was to estimate the potential cost-savings for Australian hospitals from improved surgical performance for colorectal surgery attributed to a surgical self-audit program.

**Methods:**

We used a mathematical decision-model to investigate cost differences in usual practice versus surgical audit and synthesized published hospital cost data with epidemiological evidence of adverse surgical events in Australia and New Zealand. A systematic literature review was undertaken to assess post-operative outcomes from colorectal surgery and effectiveness of surgical audit. Results were subjected to both one-way and probabilistic sensitivity analyses to address uncertainty in model parameters.

**Results:**

If surgical self-audit facilitated the reduction of adverse surgical events by half those currently reported for colorectal cancer surgery, the potential cost-savings to hospitals is AU$48,720 (95% CI: $18,080-$89,260) for each surgeon treating 20 cases per year. A smaller 25% reduction in adverse events produced cost-savings of AU$24,960 per surgeon (95%CI: $1,980-$62,980). Potential hospital savings for all operative colorectal cancer cases was estimated at AU$30.3 million each year.

**Conclusions:**

Surgical self-audit has the potential to create substantial hospital cost-savings for colorectal cancer surgery in Australia when considering the widespread incidence of this disease. The study is limited by the current availability and quality of data estimates abstracted from the published literature. Further evidence on the effectiveness of self-audit is required to substantiate these findings.

## Background

An 'adverse event' is defined as the unintentional harm arising to patients from an episode of health care and not due to the disease process itself [1]. Adverse post-operative events are a function of the surgeon's skill and judgement, the health care team in which he/she operates, patient factors such as age and presence of comorbidities, elective versus emergency presentations and hospital systems of care. Common types of adverse surgical events include surgical site infections, anastomotic leakage, deep vein thrombosis (DVT), respiratory problems such as pneumonia and pulmonary embolism, unplanned return to operating theatre, extended hospital stay, operative and 30-day mortality. Rates of adverse surgical events may be reduced by various approaches including using prophylactic antibiotics (for wound infections) and anticoagulants (for DVT), hospital infection control programs, and through surgical audits, the ongoing monitoring of outcomes and training of surgeons to improve techniques and judgements.

Surgical audit is a quality improvement process considered integral to patient care and outcomes and for the ongoing professional development of surgeons. Royal Colleges of Surgeons in Australia and the UK are firmly committed to and strongly endorse the daily practice of auditing in its various forms within a supportive, legally protective environment [2]. Ideally, surgical audit would be implemented with a modern medical record system, effective training and protected time [3]. Optimal practice also requires a closed loop, that is, an audit is performed, peer review discussion follows, learning/improved practice initiated and the steps are repeated continuously. Aside from better patient outcomes, surgical audit has other potential benefits including higher professional satisfaction, enhanced communication among colleagues and improved hospital cost-efficiencies arising from lower resource consumption [2]. However, routine use of surgical audit may not be widespread in Australia [2]. Barriers to auditing practice have been identified and include the lack of resources, lack of expertise in design and analysis, staffing communication problems and institutional impediments [3]. However, recent guidelines for surgical audit in Australia and New Zealand (NZ) have been published to help facilitate and standardise practice [1].

Computerized audit systems with dedicated software offer several advantages for auditing where surgeons or hospital staff can enter the required data, produce reports and graphs of outcomes quickly, update their cumulated cases over time, perform risk-adjusted assessments of outcomes and have the ability to pool case reports anonymously within surgical units, regions or nationally [4,5]. In comparison to conducting large surveys or labour-intensive medical chart reviews retrospectively by hospital or research staff, the routine reporting of audit data electronically is likely to facilitate a cost-effective auditing approach.

The financial toll to hospitals and health care systems for adverse surgical events is substantial primarily due to the attendant high costs incurred for extended hospital stays [6]. An additional AU $6,826 for each hospital admission was estimated, on average, in Victoria, Australia for in-patients with an adverse event, after adjusting for age and comorbidity [6]. Although many studies have investigated the costs of wound and other hospital-acquired infections, these findings are difficult to summarise due to limitations in the way methods were reported, variations in surgical type, populations, study designs and health care financing systems [7]. Nevertheless, costs per patient with surgical site infections were estimated to be approximately double those for patients without infections [7].

The purpose of this study was to estimate the potential cost-savings relating to improved surgical outcomes for colorectal surgery attributed to a computerised self-audit program. We used a modelling approach to synthesize published hospital cost data with epidemiological evidence of adverse surgical events occurring in Australasia.

## Methods

**Figure 1:**
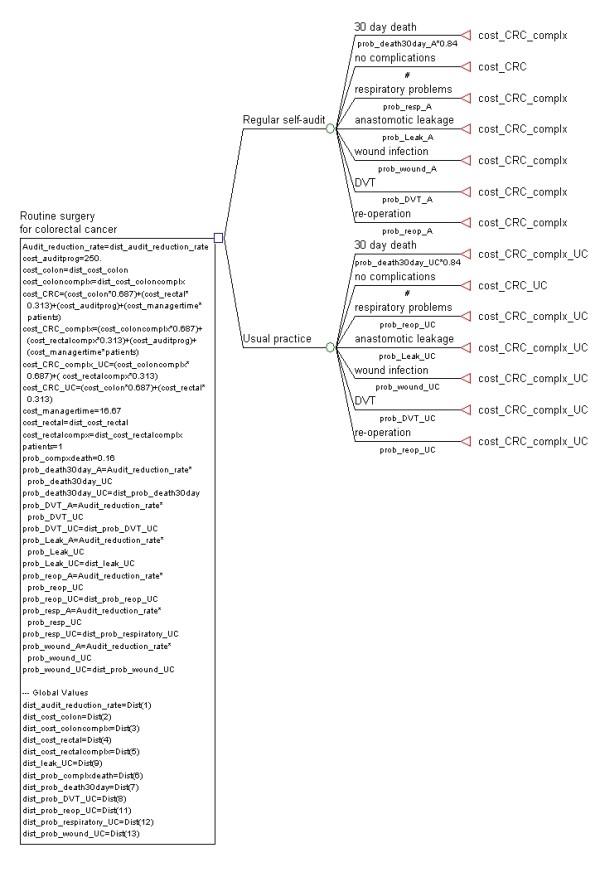
**Decision-analytical model of a hypothetical self-audit program and usual practice for surgery for colorectal cancer**.

A decision-analytical model was constructed in TreeAge Pro 2009 software (TreeAge Software Inc, Williamstown, MA, USA) (Figure 1). Two strategies were compared in the model; 'self-audit program' and 'usual practice'. The model assumed that after a period of time following continuous self-audit practice, lower rates of adverse events would eventuate and incur lower hospital costs than for usual practice. Therefore, the difference between the two groups would represent the expected cost-savings attributed to a self-audit program. The analytical time-frame was 12 months which was chosen to capture various caseloads per surgeon.

Several steps were involved in populating the model parameters and analysis. First, we identified the common types and frequencies of surgical adverse events relating to colorectal cancer in Australia and NZ along with evidence on the effectiveness of surgical audit for colorectal cancer surgery. A systematic literature review was undertaken to locate Australian and NZ studies reporting post-operative outcomes for a series of patients undergoing surgery for colorectal cancer. Studies were included if they were published after 2000 (to reflect current practice), had observational study designs and involved surgeries for colorectal cancer patients where post-operative outcomes were reported. PubMed and Medline databases were used to search for journal articles in the English language with the following terms in their title: colorectal, colon, rectal, surgery, surgical or management combined with adverse events, complications, post-operative, morbidity, performance, outcomes. A selection of the most commonly reported adverse events were included in the model; anastomotic leakage, wound infection, DVT, respiratory problems (defined as a collective group including pneumonia, other infections, pulmonary embolism), re-operation and 30-day mortality (including operative mortality). Although 'length of stay' in hospital was also a common outcome reported, this is a consequence of arising complications, it is implicit in the valuation of costs (rather than an adverse event *per se*) and therefore was not included as a separate outcome. Finally, '30-day mortality' was included in our model as the surgical literature indicates that a proportion of these are likely to be attributed to adverse events and omitting them would otherwise underestimate costs of adverse events.

For evidence relating to the effectiveness of surgical audit, the following search terms were used: colorectal, colon, rectal, surgery combined with surgical audit, surgical performance, quality, and appraisal. However, as so few studies were identified, our criteria was amended to include studies from any country. Hand-searching article references and conference abstracts were undertaken to identify additional studies. The specific search results are available in Additional file 1.

Second, costs were assigned to each branch of the two options. This cost-analysis was undertaken from a hospital perspective (both public and private) and included inpatient costs for colon and rectal surgical admissions with or without complications. Costs for surgical admissions were derived from the National Hospital Cost Data Collection 2006-2007 [8] listing Australian-Refined Diagnostic Related Groups (AR-DRG) recorded for standard and complicated hospital episodes. The charge of the self-audit computer software program and staff cost for data entry by a health information manager were also considered. Private patient costs or primary care costs for events occurring when patients returned into the community were omitted. Costs were inflated to 2009 Australian dollars using the health component of the Consumer Price Index.

Third, the model was calculated by summing the expected (mean) cost values at each tree node for each type of adverse event for the 'self-audit' and 'usual practice' arms and aggregated separately. These values are based on the probabilities of the adverse events derived from the literature and their attendant costs. An expected mean cost for one surgical episode in each arm was produced. The cost-savings arise from the reduced level of the 'surgery plus complications' DRG cost estimates in the audit arm compared to the usual practice arm. However, as the cost of adverse events is embedded in the DRG estimates and intrinsically linked to the surgical episode where there is uncertainty in the exact level of resources consumed, sensitivity analyses on the DRG cost estimates were performed. One-way sensitivity analyses on all cost and epidemiological parameters were undertaken to investigate the robustness of the base results to plausible changes in the data estimates (see Table 1 for the range of estimates tested). A probabilistic sensitivity analysis was also performed, re-sampling from nominated distributions of data inputs through 2,000 Monte Carlo simulations. Beta distributions were assigned to probability estimates (e.g., adverse event rates, effectiveness rate of surgical audit). Gamma distributions were assigned to surgery cost variables because in practice these are normally right-skewed [9] (e.g., in the case of persistent complications and associated long hospital stay). The mean incremental costs and 95% confidence intervals were extrapolated for different surgical caseloads typically performed by surgeons in Australia and NZ to obtain the total cost-savings possible over 12 months. The 95% confidence intervals reflect the different combinations of plausible adverse event rates in the literature and variations in cost. Finally, the results were calculated for all colorectal surgeries undertaken in Australia during one year.

**Table 1:** Data parameters used in calculations, plausible ranges, sources and assumptions

Description	Estimate (plausible range)	Source
Surgical cases per year (for one surgeon)	Scenario 1: 20^1^	[11](<7, ≥7, over 15 per 3 months)
	Scenario 2: 40	
	Scenario 3: 70	

% reduction in adverse event rates	Scenario 1: 50% (base effect)	[4,17,22-24,30,31]
	Scenario 2: 25% (small effect)	
	Scenario 3: 75% (large effect)	

% of colon and rectal surgery cases		
Colon	68.7-75.9%	[11]
Rectal	24.1-31.3%	

Baseline adverse event rates^2,3^		
Anastomotic leak	4.4% (0.5-8.2)	Mean of low/high values from up to 11 studies [4,10-19,22] (see Table 2)
Wound infection	5.6% (2.1-9.1%)	
DVT	3.5% (0.3-6.7%)	
Respiratory complications (pulmonary embolism, infection, pneumonia)	5.5% (0.2-10.7%)	
Return to operating theatre	7.5% (2.7-12.2%)	

Post-op deaths (<30 days) %	3.3% (0.2-6.4%)	As above
(% attributed to complications)	(16%)	[11]

Hospital costs (AUD 2009):		AR-DRGs code (ALOS):
Rectal resection with complications	$33,277	G01A (18.4 days)
Rectal resection with no complications	$18,094	G01B (9.8 days)
Colon procedures with complications	$30,899	G02A (17.8 days)
Colon procedures with no complications	$14,283	G02B (8.2 days)

12-month subscription to 'surgical performance' self-audit software^4^	$250	http://www.surgicalperformance.com US$200

Data entry of surgical outcomes into audit software^5 ^- performed by health information manager, 20 minutes per audit	$16.67 per audit	Based on salary $50 per hour

Abbreviations: ALOS - average length of stay, DVT - deep vein thrombosis, AR-DRG - Australian Refined Diagnostic Related Group

1. Scenario 1 would apply to the majority of surgeons in Australia and New Zealand [11].

2. Implicit in the plausible range of complication rates listed above are the variations reflected in the usual population of cases requiring colon or rectal surgery, i.e., a mix of emergency and elective cases, colon and rectal cases, age, Dukes stage and presence of comorbidity.

3. As there is no evidence on extent of type of multiple complications occurring concurrently with colorectal surgery patients, the estimates assume mutually exclusive complication rates. An exception is '30-day mortality' where the percentage of 30-day deaths attributed to complications is 16% and costs were adjusted down to avoid double-counting.

4. Self-audit process involves feedback with peers - any attendant costs here were not included. It is assumed peer discussions would be periodically scheduled in normal practice.

5. Data entry is assumed to incur the time of a health info manager.

### Results

#### Evidence for adverse surgical outcomes

Eleven studies reported adverse surgical outcomes in a series of patients following surgery for colorectal cancer (Table 2). The primary goal of all studies was to document the surgical management and patient outcomes in their populations. Two reports documented outcomes for the same study [10,11]. In summary, the range of published adverse events used to populate our model were 0.5-2.8% for anastomotic leaks, 2.1-9.1% for wound infections, 0.3-6.7% for DVT, 2.7-12.2% for return to theatre, 0.2-10.7% for respiratory conditions and 0.2-7.7% for 30-day deaths. The mean of these ranges were used for the base case scenario with sensitivity analyses using the low and high values. The rates of adverse events varied considerably but the difference between the low and high rates reported were consistently around 7-9 percentage points. An exception was wound infection rates of 22.3% for series of 133 patients in Nelson, NZ that was more than double those reported elsewhere and may be explained by the researchers including both superficial and deep infections [12]. This rate was considered an outlier and omitted from our summary estimates. Five studies, including three in NZ [12-14], reported outcomes from regional areas [4,12-15] while the remainder were state-wide [16], national [10,11,17] or urban-based [18,19]. One study accounted for pre-operative factors and outcomes were risk-adjusted [4]. No study reported estimates on multiple complications occurring concurrently during the hospital stay and only two studies reported mortality rates directly arising from the complications [11,19]. Post-operative outcomes tended to be poorer for emergency presentations and for rectal surgery, the latter which involves more complex and difficult surgery [10,15]. In the National Colorectal Cancer Survey, no statistically significant differences were found for post-operative morbidity and mortality for high- and low-volume surgeons (defined as ≥7 or <7 surgeries per quarter, respectively) although some pre-operative practice differences were evident [11].

**Table 2:** Australian and NZ studies reporting adverse events from surgery involving patients with colorectal cancer

Author, Year & No. cases	% elective cases	% anastomotic leak (AL)	% wound infection	% DVT	% return to theatre	% respiratory	% 30-day deaths
Semmens 2000 [16] n = 9,673	77%	6.5%	8.3% post-op infect	ns	Most of AL (est.5%)	ns	4.2%

Birks 2001 [15] n = 877	69%	3.3%	7.2%	ns	5.7%	ns	4.1% CRC patients

Kable 2002 [17] n = 5,432	ns	ns	2.1%	0.3%	ns	pneumonia 0.2%	0.8%

Killingback 2002 [19] n = 1,418	100%	4.1%	2.1%	1.1%	2.7%	6.7% (incl various)	1.6%

McGrath 2004 [10,11] & 2005 n = 1,911	86-93%	0.0-3.0%	6.6-9.1%	1.0-6.7%	ns	0-1.6% pulmonary embolism	4.0-4.3%

Wong 2005 [30] n = 1,293	83%	0.5-1.1%	4.2-7.8%	2.3-3.9%	2.7-6.7%	ns	1.2-7.7%

Gollop 2006 [13] n = 170	71%	3%	Ns	ns	12%	ns	5%

Samson 2007 [14] n = 191	ns	4.5%	11%	ns	7%	ns	4%

O'Grady 2007 [12] n = 133	91%	4.7%	22%	ns	3%	7.5%	0.8%

Bowles 2007 [4] n = 500 pre n = 100 post	63%	Pre 8.2% Post 1.4%	Ns	ns	Pre 12.2% Post 5%	ns	Pre 6.38% Post 0%

Frye 2009 [18] n = 1,513	100%	3.8%	Ns	ns	ns	ns	0.2%

SUMMARY^1^ low-high values		0.5-8.2%	2.1-9.1%	0.3-6.7%	2.7-12.2%	0.2-10.7%	0.2-7.7%

(Abbreviations: ns = not stated, DVT = deep vein thrombosis, AL = anastomotic leak, CRC = colorectal cancer)

1. Summary rates exclude 0% where reported and 22.3% wound infection rate from O'Grady 2007 that was considered an outlier and possibly due to inclusion of superficial and deep infections.

### Evidence for effectiveness of surgical audit

The evidence-base for 'audit and feedback' interventions is very large for clinical studies across a huge range of medical topics. A Cochrane systematic review of 118 studies on this topic showed small to moderate improvements in outcomes overall (median 5%, range 3-11%) [20]. However, studies which evaluated audit programs for surgery and post-operative outcomes (for any disease) was scarce. For colorectal cancer surgery, one Australian study by Bowles 2007 involved a pre-post study design over four years showing favourable improvements following the introduction of an audit program (Table 2) [4]. For example, anastomotic leak rates decreased from 8.2% to 1.4%, re-operations from 12.2% to 5% and pneumonia from 10.7% to 8.5%. Audit and regular educational feedback within the Lothian Surgical Audit program (UK) also produced notable improvements for colorectal surgery outcomes; anastomotic leakage from 5% to 2% and stoma rates following rectal cancer surgery from 28% to 12% [21,22]. In a second Australian study involving surgery for all diseases, Kable *et al. *(2002) discuss the 'degree of preventability' of adverse surgical events [17]. A proportion of complications have low preventability (31.4%) while others were highly preventable (47.6%). As such, the authors highlight that it is not possible to fully eliminate all adverse events as 21% were not considered preventable [17]. In the Scottish Audit of Surgical Mortality, significant improvements in most adverse factors occurred over 9 years (1994-2002) after audit and peer review by surgical assessors [23]. These included failure to use DVT prophylaxis reducing from 4.6% to 0.7%, delay in recognising complications reduced from 8.2% to 6%, failure to use high-dependency or intensive care units reduced from 18.4% to 5.4% [23]. Finally, support for computerized surgical audit for colorectal cancer is advocated [5,24] although formal evaluation of their effectiveness have not been reported.

### Results of model

**Figure 2:**
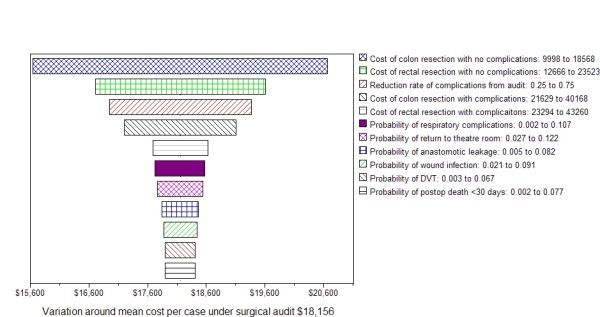
**Results of the one-way sensitivity analyses**.

The data used to populate the model included estimates reported in the published literature however a number of assumptions were also necessary (Table 1). The expected mean costs of surgical treatment for a single case of colorectal cancer, under usual practice was $20,627 compared to that for a self-audit program $18,156 if the self-audit program reduced common adverse events by half. A cost-saving of $2,471 per case was predicted. Sensitivity analysis showed that changes to this expected mean cost of $18,156 was most sensitive to variation of ± 30% in the cost of uncomplicated colon surgery (range $15,652-$20,661) and rectal surgery (range from $16,711-$19,602) (Figure 2).

Expected mean costs for the two options are more precise from a probability sensitivity analysis (Table 3). For each surgical patient, results from the probabilistic sensitivity analysis produced mean cost savings of $2,436 for a 50% reduction in adverse events, $1,248 for a 25% reduction and $3,636 for a 75% reduction (Table 3). These means and their 95% confidence intervals are extrapolated to annual caseloads of 20, 40 or 70 to estimate annual cost-savings for one surgeon (Table 3). For the most typical caseload of around 20 surgeries per year for one surgeon [11], average cost-savings were estimated at $48,720 (95%CI: $18,080-$89,260). The annual incidence of colorectal cancer in Australia 2005 was 13,076 cases. Assuming 95% of all cases have surgery and calculating the potential cost-savings of $2,436 for each person (on average) if adverse event rates are halved, it is estimated that Australian hospitals could save $30.3 million dollars per year.

**Table 3:** Potential annual cost-savings for reduced adverse events for colorectal cancer surgery by surgical caseload (AU$ 2009)

% reduction in adverse events	No. cases	Mean cost-saving $	(95% CI) $
50% (baseline)	1	2,436	904 - 4,463
	20	48,720	18,080 - 89,260
	40	97,440	36,160 - 178,520
	70	170,520	63,280 - 312,410

25% (small effect)	1	1,248	99 - 3,149
	20	24,960	1,980 - 62,980
	40	49,920	3,960 - 125,960
	70	87,360	6,930 - 220,430

75% (large effect)	1	3,636	1,580 - 6,047
	20	72,720	31,600 - 120,940
	40	145,440	63,200 - 241,880
	70	254,520	110,600 - 423,290

## Discussion

We used a mathematical model to synthesize the published evidence on the extent of surgical adverse events and attendant costs for colorectal cancer surgery in Australia. The advantage of this type of model is that it explicitly identifies the options and parameters under consideration and allows for detailed sensitivity and scenario (what-if) analyses. Our findings showed that if self-audit facilitated the reduction of adverse surgical events by half those currently reported for colorectal cancer surgery, the potential annual hospital cost-savings was $48,000 for each surgeon treating 20 cases per year and could range from $18,000 to $90,000. The wide range of potential cost-savings reflects the combined uncertainty of published estimates on adverse events rates, different surgical casemix and the potential variation in the accompanying costs. The potential cost-savings is substantial when considering the widespread incidence of this disease and the cost containment goals in all hospitals.

The study is limited by the current availability and quality of data estimates abstracted from the published literature. The 11 studies providing estimates on adverse event rates were inconsistent in the use of standard definitions and methods used to measure the various types of adverse events. This appears to be a common problem internationally as confirmed in a large UK review on the topic of monitoring adverse events [25]. The current Australian and NZ guidelines for surgical audit may alleviate these problems [1]. Furthermore, each study used a different set of outcomes, despite assessing the same disease (colorectal cancer), and few have adjusted for baseline (pre-operative) risk characteristics (e.g., age, comorbidities, body mass index). In addition, it is evident that differences exist in the case mix of patients in the studies, with some studies having a higher proportion of emergency presentations [4,13,15]. Despite this, the adverse event rates did not always appear higher than for the group of studies overall, as may have been expected. Nevertheless, comparisons across studies are problematic. It has also been stated that studies involving surveys of surgeons with self-reported data or retrospective medical chart reviews are likely to underestimate the true prevalence of adverse events and may be unreliable [11].

The lack of evidence on the effectiveness level of audit programs for surgery is also a limitation of the study. This is probably because of, in part, the confronting nature of the topic, the possible legal and social ramifications, time pressures and overall reluctance of doctors to be involved in this kind of study. As assumptions were made about this effect size, the findings should be considered as exploratory. Furthermore, the effectiveness level of surgical audit is governed, in part, by the initial adverse event rates with greater improvements possible when rates are relatively high to begin with [4,23]. However, our findings are based on the best available evidence and provide an indication of the level of cost-savings possible. A more robust analysis would require evidence from a prospective randomized intervention trial that evaluates the potential health benefits and precise cost-savings of self-audit benefits in comparison with usual practice. It would be important for surgical outcomes to be risk-adjusted [26] using a validated system like CUSUM [4]. However, such a trial may be unethical and not particularly policy-changing since The Royal Australian College of Surgeons already encourages systematic auditing practice[1].

The analysis also relied on an aggregated national costing report on episodes of colorectal surgery in Australian hospitals [8]. While there are studies reporting costs of hospital-acquired surgical site infections [27,28], for consistency across the other outcome types, a generic AR-DRG cost (with complications) was assigned. A more detailed micro-costing approach may have provided precise estimates for each complication type but is very resource-intensive and uncommon [29]. Furthermore, micro-costing may have produced higher costs per person with an adverse event, as previously found for surgical site infections [28], and would imply that our average cost-savings may be underestimated, but conservative. Furthermore, our costs are likely to a fraction of all health care costs because we have excluded any costs flowing on to primary care and community health providers. For example, several studies have confirmed that more than 50% of surgical site infections occurred post-discharge creating substantial and more hidden costs eventually borne by primary care providers [27,28].

## Conclusions

Using best-available evidence on the outcomes of colorectal cancer surgery in Australia and NZ and exploring the cost-savings accompanying lower hospital resource consumption from improvements in surgical performance, we found substantial economic dividends accruing for hospitals. A computerised self-audit program operating with frequent data input is a low-cost tool to facilitate potential improvements in surgical performance.

## Abbreviations

AR-DRG: Australian-Refined Diagnostic Related Group; AU: Australian; CI: Confidence interval; CUSUM: Cumulative sum analysis; DVT: Deep vein thrombosis; NZ: New Zealand.

## Competing interests

L Gordon received an honorarium from A Obermair for undertaking the analysis. A Obermair has a financial interest in the online self-audit software mentioned in this article and available at http://www.surgicalperformance.com.

## Authors' contributions

LG performed the data analyses and drafted the manuscript. AO assisted with interpretation, presentation of the findings and provided clinical expertise and input into the overall findings. Both authors have contributed substantively to writing the manuscript and have approved the final version.

## Authors' information

L Gordon is a health economist with a special interest in the area of cancer and population health research. A Obermair holds clinical research (chief investigator) roles in areas of surgery in gynaecology oncology, projects that are funded through the National Health and Medical Research Council.

## Pre-publication history

The pre-publication history for this paper can be accessed here:

http://www.biomedcentral.com/1471-2482/10/4/prepub

## Supplementary Material

Additional file 1 **Review studies reporting on adverse surgical outcomes following surgery for colorectal cancer**. Tabulated results of the systematic review on studies reporting on adverse surgical outcomes following surgery for colorectal cancer. Click here for file
