# Supplementary material for: Potential hospital cost-savings attributed to improvements in outcomes for colorectal cancer surgery following self-audit
Source: BMC Surg. 2010 Jan 27;10:4. doi: 10.1186/1471-2482-10-4 (PMC2835671; doi:10.1186/1471-2482-10-4)
Supplement: Additional file 1 — Review studies reporting on adverse surgical outcomes following surgery for colorectal cancer. Tabulated results of the systematic review on studies reporting on adverse surgical outcomes following surgery for colorectal cancer. [file 1471-2482-10-4-S1.DOCX]

**Supplementary file: Search results from systematic reviews**

Database: MEDLINE

Limits: title, 2000+, journal article

| Search terms | Hits | Discarded | Kept |
| --- | --- | --- | --- |
| Colorectal AND adverse AND events AND Australia* | 1 | 1 | 0 |
| Colorectal AND surgery AND Australia* | 30 | 28 | 2 [1, 2] |
| Colorectal AND surgical AND Australia* | 7 | 6 | 1 [3] |
| Colorectal AND outcomes AND Australia* | 14 | 11, 1 duplicate [2] | 2 [4, 5] |
| Colorectal AND morbidity AND Australia* | 1 | 0 | 1 [6] |
| Colorectal AND postoperative AND Australia* | 0 |  |  |
| Colorectal AND complications AND Australia* | 0 |  |  |
| Colorectal AND performance AND Australia* | 0 |  |  |
| Colorectal AND management AND Australia* | 3 | 0 | 3 [7-9] |
|  |  |  |  |
| Colon AND adverse AND events AND Australia* | 0 |  |  |
| Colon AND surgery AND Australia* | 0 |  |  |
| Colon AND surgical AND Australia* | 4 | 4 | 0 |
| Colon AND outcomes AND Australia* | 0 |  |  |
| Colon AND morbidity AND Australia* | 0 |  |  |
| Colon AND postoperative AND Australia* | 0 |  |  |
| Colon AND complications AND Australia* | 1 | 1 | 0 |
| Colon AND performance AND Australia* | 0 |  |  |
|  |  |  |  |
| Rectal AND adverse AND events AND Australia* | 0 |  |  |
| Rectal AND surgery AND Australia* | 5 | 0 |  |
| Rectal AND surgical AND Australia* | 1 | 0 |  |
| Rectal AND outcomes AND Australia* | 0 |  |  |
| Rectal AND morbidity AND Australia* | 3 |  |  |
| Rectal AND postoperative AND Australia* | 1 | 0 |  |
| Rectal AND complications AND Australia* | 0 |  |  |
| Rectal AND performance AND Australia* | 0 |  |  |
|  |  |  |  |
| Colorectal AND audit AND Australia* | 5 | 2, 2 duplicates [1, 6] | 1 [10] |
| Surgical AND audit AND Australia* | 11 | 11 | 0 |
| Surgical AND appraisal | 0 |  |  |
| Surgical AND adverse AND events | 26 | 25 | 1 [11] |

Database: PubMED

Limits: title, 2000+, English, journal article

| Search terms | Hits | Discarded | Kept |
| --- | --- | --- | --- |
| Colorectal AND adverse AND events AND Australia* | 21 | 21 | 0 |
| Colorectal AND surgery AND Australia* | 3 | 1, 2 duplicates [1, 2] | 0 |
| Colorectal AND surgical AND Australia* | 0 |  |  |
| Colorectal AND outcomes AND Australia* | 1 | 1 duplicate [2] | 0 |
| Colorectal AND morbidity AND Australia* | 0 |  |  |
| Colorectal AND postoperative AND Australia* | 0 |  |  |
| Colorectal AND complications AND Australia* | 0 |  |  |
| Colorectal AND performance AND Australia* | 0 |  |  |
| Colorectal AND New Zealand | 14 | 12, 2 duplicates [5, 9] | 0 |
|  |  |  |  |
| Colon AND adverse AND events AND Australia* | 0 |  |  |
| Colon AND surgery AND Australia* | 0 |  |  |
| Colon AND surgical AND Australia* | 0 |  |  |
| Colon AND outcomes AND Australia* | 0 |  |  |
| Colon AND morbidity AND Australia* | 0 |  |  |
| Colon AND postoperative AND Australia* | 0 |  |  |
| Colon AND complications AND Australia* | 0 |  |  |
| Colon AND performance AND Australia* | 0 |  |  |
| Colon AND New Zealand | 4 | 4 | 0 |
|  |  |  |  |
| Rectal AND adverse AND events AND Australia* | 0 |  |  |
| Rectal AND surgery AND Australia* | 0 |  |  |
| Rectal AND surgical AND Australia* | 0 |  |  |
| Rectal AND outcomes AND Australia* | 0 |  |  |
| Rectal AND morbidity AND Australia* | 0 |  |  |
| Rectal AND postoperative AND Australia* | 0 |  |  |
| Rectal AND complications AND Australia* | 0 |  |  |
| Rectal AND performance AND Australia* | 0 |  |  |
| Rectal AND New Zealand | 2 | 2 | 0 |
|  |  |  |  |
| Colorectal AND audit | 32 | 30, 1 duplicate [1] | 1 [12] |
| Surgical AND audit | 35 | 34 | 1 [13] |
| Surgical AND appraisal | 9 | 9 | 0 |
| Surgical AND adverse AND events | 1 | 1 | 0 |
| Computerised AND audit AND colorectal | 1 | 0 | 1 [14] |
| Computerized AND audit AND colorectal | 0 |  |  |

Hand-searching references, scanning ANZ J Surg table of contents– uncovered 4 additional studies [15-18]1 [17]

**References**

1. Birks DM, Gunn IF, Birks RG, Strasser RP: **Colorectal surgery in rural Australia: scars; a surgeon-based audit of workload and standards**. *ANZ J Surg* 2001, **71**(3):154-158.

2. Semmens JB, Platell C, Threlfall TJ, Holman CD: **A population-based study of the incidence, mortality and outcomes in patients following surgery for colorectal cancer in Western Australia**. *Aust N Z J Surg* 2000, **70**(1):11-18.

3. Wong SK, Kneebone A, Morgan M, Henderson CJ, Morgan A, Jalaludin B: **Surgical management of colorectal cancer in south-western Sydney 1997-2001: a prospective series of 1293 unselected cases from six public hospitals**. *ANZ J Surg* 2005, **75**(9):776-782.

4. Bowles TA, Watters DA: **Time to CUSUM: simplified reporting of outcomes in colorectal surgery**. *ANZ J Surg* 2007, **77**(7):587-591.

5. Samson PB, Ngaei G: **Colorectal resection in peripheral New Zealand: workload, outcomes and its future**. *ANZ J Surg* 2007, **77**(11):999-1003.

6. Killingback M, Barron P, Dent O: **Elective resection and anastomosis for colorectal cancer: a prospective audit of mortality and morbidity 1976-1998**. *ANZ J Surg* 2002, **72**(10):689-698.

7. McGrath DR, Leong DC, Armstrong BK, Spigelman AD: **Management of colorectal cancer patients in Australia: the National Colorectal Cancer Care Survey**. *ANZ J Surg* 2004, **74**(1-2):55-64.

8. McGrath DR, Leong DC, Gibberd R, Armstrong B, Spigelman AD: **Surgeon and hospital volume and the management of colorectal cancer patients in Australia**. *ANZ J Surg* 2005, **75**(10):901-910.

9. O'Grady G, Secker A: **Colorectal cancer management in the provincial New Zealand setting of Nelson**. *ANZ J Surg* 2007, **77**(11):1004-1008.

10. Gollop SJ, Fancourt MW, Gilkison WT, Kyle SM, Mosquera DA: **Prospective audit of colorectal resections in a peripheral public hospital**. *ANZ J Surg* 2006, **76**(9):817-820.

11. Kable AK, Gibberd RW, Spigelman AD: **Adverse events in surgical patients in Australia**. *Int J Qual Health Care* 2002, **14**(4):269-276.

12. Ugolini G, Rosati G, Montroni I, Zanotti S, Manaresi A, Giampaolo L, Taffurelli M, Pricolo V: **An easy-to-use solution for clinical audit in colorectal cancer surgery**. *Surgery* 2009, **145**(1):86-92.

13. Thompson AM, Ashraf Z, Burton H, Stonebridge PA: **Mapping changes in surgical mortality over 9 years by peer review audit**. *Br J Surg* 2005, **92**(11):1449-1452.

14. Harriss DR, Blake JR: **Computerised audit for colorectal cancer**. *Ann R Coll Surg Engl* 1993, **75**(4):268-271.

15. **Lothian and Borders large bowel cancer project: immediate outcome after surgery. The consultant surgeons and pathologists of the Lothian and Borders Health Boards**. *Br J Surg* 1995, **82**(7):888-890.

16. Aitken RJ, Nixon SJ, Ruckley CV: **Lothian surgical audit: a 15-year experience of improvement in surgical practice through regional computerised audit**. *Lancet* 1997, **350**(9080):800-804.

17. Frye J, Bokey EL, Chapuis PH, Sinclair G, Dent OF: **Anastomotic leakage after resection of colorectal cancer generates prodigious use of hospital resources**. *Colorectal Dis* 2008, **28**:28.

18. Marson LP, Stevenson J, Gould A, Aitken RJ: **A prospective colorectal cancer audit appears to be improving outcome.** *Br J Surg* 1997, **84 (Suppl 1)**:24.
